# Update on esophageal function, acid and non-acid reflux after one-anastomosis gastric bypass (OAGB): high-resolution manometry, impedance-24-h pH-metry, and gastroscopy in a prospective mid-term study

**DOI:** 10.1007/s00464-025-11606-7

**Published:** 2025-02-18

**Authors:** D. M. Felsenreich, N. Vock, M. L. Zach, I. Kristo, J. Jedamzik, C. Bichler, J. Eichelter, M. Mairinger, L. Gensthaler, L. Nixdorf, P. Richwien, L. Pedarnig, F. B. Langer, G. Prager

**Affiliations:** Division of Visceral Surgery, Department of General Surgery, Medical University of Vienna, Waehringer Guertel 18-20, 1090 Vienna, Austria

**Keywords:** One-anastomosis gastric bypass, Impedance-24-h pH-metry, High-resolution manometry, Gastroscopy, GERD, Quality of life

## Abstract

**Background:**

One-anastomosis gastric bypass (OAGB) is the third most common metabolic/bariatric procedure worldwide. A point for discussion regarding OAGB is acid and non-acid reflux in mid- and long-term follow-up. The aim of this study was to objectively evaluate reflux and esophagus motility by comparing pre- and postoperative results of 24-h pH-metry, high-resolution manometry (HRM), and gastroscopy.

**Setting:**

Cross-sectional study and university hospital based.

**Methods:**

This study includes primary OAGB patients operated at the Medical University of Vienna before 31st December 2022. After a mean follow-up of 4.1 ± 2.9 years, the preoperative examinations were repeated. Additionally, history of weight, remission of obesity-related complications (ORC), and quality of life (QOL) were evaluated.

**Results:**

A total of 50 patients were included in this study and went through all examinations. Preoperative weight was 125.5 ± 21.0 kg with a BMI of 44.6 ± 5.4 kg/m^2^ and total weight loss after 4.1 ± 2.9 years was 37.1 ± 8.1%. Remission of ORC and QOL outcomes was successful in all categories. Gastroscopy showed anastomositis, esophagitis, Barrett’s esophagus, and bile in the pouch in 38.0%, 34.0%, 6.0%, and 48.0%, respectively. In HRM, the postoperative lower esophageal sphincter pressure was 29.6 ± 15.1 mmHg (unchanged to preoperative). The total number of refluxes was equal to preoperative, whereas decreased acid refluxes were replaced by increasing non-acid refluxes. Impedance-24-h pH-metry showed that acid exposure time of the esophagus and DeMeester score decreased significantly to 1.6 ± 1.4% (*p* = 0.001) and 10.3 ± 9.6 (*p* = 0.046).

**Conclusion:**

This study has shown decreased rates of acid reflux and increased rates of non-acid reflux after a mid-term outcome of primary OAGB patients. Gastroscopy showed significant signs of chronic reflux exposure of the anastomosis, the pouch, and the distal esophagus, even in asymptomatic patients. General follow-up visits in patients after OAGB should be considered.

**Graphical abstract:**

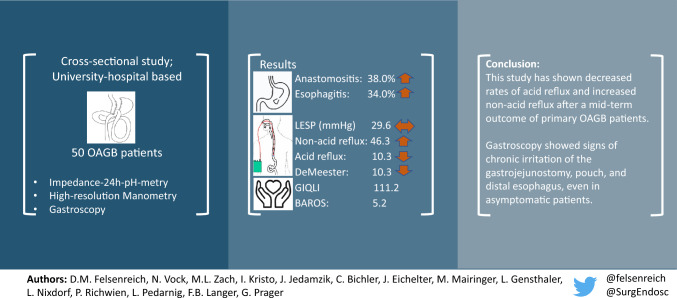

Severe obesity and obesity-related complications (ORC) of the metabolic syndrome are constantly increasing worldwide [1, 2]. Affected patients do not only have a decreased quality of life (QOL) but also a lower life expectancy [3, 4]. Currently, metabolic/bariatric surgery (MBS) is the most effective treatment for long-lasting weight loss and remission/improvement of obesity-related complications (ORC) [5]. One-anastomosis gastric bypass (OAGB) is the third most common bariatric procedure worldwide with increasing numbers of performances each year. OAGB is recognized and approved by the International Federation for the Surgery of Obesity and Metabolic Disorders (IFSO) [6, 7].

Basic points in surgical technique of OAGB are a long and narrow pouch, a biliopancreatic limb (BPL) of 150–175 cm, and anti-reflux-sutures where the BPL is attached to the patient’s left side of the distal pouch [8]. In contrast to the standard Roux-en-Y Gastric Bypass (RYGB), no jejuno-jejunostomy is performed [9]. The advantages of OAGB are a successful weight loss as well as remission/improvement of ORC [10]. One of the disadvantages of OAGB is a potential risk of biliary reflux, reports of which range from 0.7 to 7.6% of affected patients in the literature [11, 12].

Studies available in the literature at this time mainly focus on symptoms of biliary reflux, conversion rates to RYGB, and results from gastroscopies [11, 13, 14]. Only a few studies with low numbers of patients and short follow-up currently report objectively measured data, such as impedance-24-h pH-metry or high-resolution manometry (HRM) [15, 16].

The present study is an update of the publication by Felsenreich et al. [17] where the authors were able to more than double the number of included patients performing functional testing of the esophagus before and after OAGB. This makes it the largest series of objective testing in a mid-term follow-up comparing pre- and postoperative mid-term results after OAGB.

The aim of this study update was to evaluate acid and non-acid reflux as well as esophagus motility, comparing results from impedance 24-h pH-metry, HRM, and gastroscopy before and after OAGB in a mid-term follow-up. Further, weight loss, remission of ORCs, and QOL after OAGB were evaluated.

## Patients and methods

A total of 969 patients with severe obesity received a primary OAGB at the Medical University of Vienna before 31st December 2022. Revisional OAGB patients were not considered for this study. Of these 969 patients, 172 had complete sets of the following preoperative examinations: gastroscopy, impedance-24-h pH-metry, HRM. For this study, 50 patients were willing to repeat these examinations postoperatively. In addition, pre- and postoperative data on patients’ weight, ORC, GERD, and QOL were compared.

Patients with metabolic/bariatric procedures before the OAGB, postoperative complications, and reoperations were not included in this study as this could compromise the quality of data gathered. Additionally, none of the patients in this study were suffering from clinical GERD before the OAGB: GERD is a contraindication to OAGB in our metabolic/bariatric center. The hiatus was checked for hiatal hernias in the preoperative gastroscopy and intraoperatively and then repaired by posterior hiatoplasty if indicated.

The mean follow-up in this study was 4.1 ± 2.9 years, with a minimal follow-up of 1 year. The median follow-up was 4.2 (range 1.1–6.9) years. The follow-up rates for the examinations were 100% (50/50 patients) for gastroscopy, 100% (50/50 patients) for impedance-24-h pH-metry, 100% (50/50 patients) for HRM, and 68% (34/50 patients) for the QOL questionnaires.

The local ethical committee of the Medical University of Vienna has approved this follow-up study (reference number: 1752/2019) and an informed consent was obtained from all participating patients.

### Surgical technique

The surgical technique of OAGB was standardized in all patients and previously described in detail [8]. Thus, only some brief facts are included here, starting after the trocars are placed. At this point, the lesser sac (bursa omentalis) is opened at the smaller curvature of the stomach about 1–2 cm below the incisura angularis (area of the crow’s feet). Then, the stomach is partially transected horizontally with a 45-mm stapler. Next, the left crus of the diaphragm is dissected free. In patients with a preoperatively detected hiatal hernia in the gastroscopy, both crura are visualized. First, the hiatal hernia is verified, a posterior hiatoplasty (and anterior, if necessary) is performed after complete dissection and mobilization of the distal esophagus.

Next, a 12-mm (36 French) bougie is introduced and a long and narrow pouch created using four 60-mm stapler cartridges. The angle of Treitz is identified and 150 cm (up to 250 cm in this collective) of the BPL is measured to create a linear end-to-side gastro-jejunostomy using a 30-mm cartridge. Then, a non-absorbable running suture is placed between the end of the BPL and the distal 6 cm of the vertical staple line of the pouch in order to create an “anti-reflux” mechanism. Further steps are the closure of the Petersen’s defect and a non-absorbable single-knot suture from the alimentary limb to the antrum of the remnant stomach to prevent kinking of the alimentary limb. This should avoid pooling of bile in the pouch, which can lead to biliary reflux. Finally, an air-leakage test of the gastro-jejunostomy is performed with continuous air insufflation by gastroscopy.

### History of weight- and obesity-related complications

Patients’ weight and BMI were examined at the time of the OAGB, at nadir (lowest postoperative weight), and at the time of the follow-up. Excess weight loss (%EWL), total weight loss (%TWL), and the change in BMI were calculated.

Further, existing ORC (based on intake of medication) at the time of the OAGB and at follow-up were evaluated. These were type 2 diabetes mellitus (T2D), arterial hypertension, obstructive sleep apnea syndrome (OSA), and hyperlipidemia. Additionally, data on GERD symptoms and the need for proton pump inhibitor intake were recorded.

### Gastroscopy

As a standard in our center, all patients had gastroscopy before the OAGB. In all examinations, standardized biopsies from the antrum, corpus, and the gastro-esophageal junction were taken. The latter were done according to the Seattle protocol [18]. The diagnosis of Barrett’s esophagus was performed according to the American Gastroenterologist Association (AGA), meaning the detection of goblet cells, intestinal metaplasia, and macroscopic detection of column line esophagus (CLE) [19]. Hiatal hernias and all other saliences were noted. If *Helicobacter pylori* was detected, preoperative eradication was done in all patients.

Postoperative follow-up gastroscopies were performed specifically for this study. Biopsies from the anastomosis, the pouch, and again from the gastro-esophageal junction were performed. A specific focus was set on the detection of bile in the pouch/esophagus and of anastomotic ulcers. All other pathological issues were recorded as well.

### High-resolution manometry

HRM was similar in the pre- and postoperative setting, using a solid-state catheter with 32 circumferential pressure sensors. The parameters were assessed with ten 5-ml liquid swallows at intervals of 30 s. Outcomes were the lower esophageal sphincter pressure (LESP) and the transit-time of a liquid bolus. Further, the distal contractile integral (DCI) and the integrated relaxation pressure (IRP) were measured. These measurements represent the power of the peristaltic waves of the esophagus during bolus transit and the lowest pressure at the gastrointestinal junction during opening [20]. Available reference levels are listed in Table 4.

### Impedance-24-h pH-metry

Immediately after the HRM was done, the probe for impedance-24-h pH-metry was placed in the distal esophagus and left for 24 h. Again, the pre- and postoperative settings of this examination were the same. First, the acid exposure time was measured, which implies the percentage of time in 24 h; the pH value in the distal esophagus is below 4. Second, the total number of reflux episodes in 24 h from the stomach or pouch to the esophagus was counted. These can be divided into acid and non-acid refluxes. High numbers of non-acid refluxes may reflect biliary reflux. Finally, the DeMeester score is calculated based on duration and number of acid refluxes in different body positions during 24 h [21]. Again, the reference levels are listed in Table 4.

### Questionnaires

Patients in this study were also asked to complete four questionnaires at the time of the follow-up: Short Form-36 (SF-36), Gastrointestinal Quality of Life Index (GIQLI), Bariatric Quality of Life index (BQL), and Bariatric Analysis and Reporting Outcome System (BAROS). Preoperative questionnaire results were not available as they were not gathered in a standardized manner at the time of the primary operation.

SF-36 is a validated questionnaire examining patients’ general QOL in 36 items. In eight sections, questions about physical and psychological health are raised, where a score between 0 and 100 can be reached [22].

GIQLI was developed by Eypasch et al. in 1993 and consists of 36 questions with a total score of 144 points. This questionnaire focuses on different types of gastrointestinal symptoms, including GERD, flatulence, abdominal pain, bowel movement, and diarrhea [23].

BQL is a practical tool to evaluate QOL in patients undergoing metabolic/bariatric surgery. It was developed in 2005 and updated in 2009 by Weiner et al. and consists of 13 questions, with a total of 65 points equaling 100% [24].

BAROS is an outcome score to evaluate the success of a metabolic/bariatric procedure. Beside weight loss, QOL, remission, and improvement of ORC, complications and reoperations are evaluated as well. Then, the outcome can be classified as failure, fair, good, very good, or excellent [25].

### Statistical analysis

Due to the descriptive nature of this study, a power analysis was not appropriate. All patients suitable with the inclusion criteria were considered for participation in this study.

**Fig. 1:**
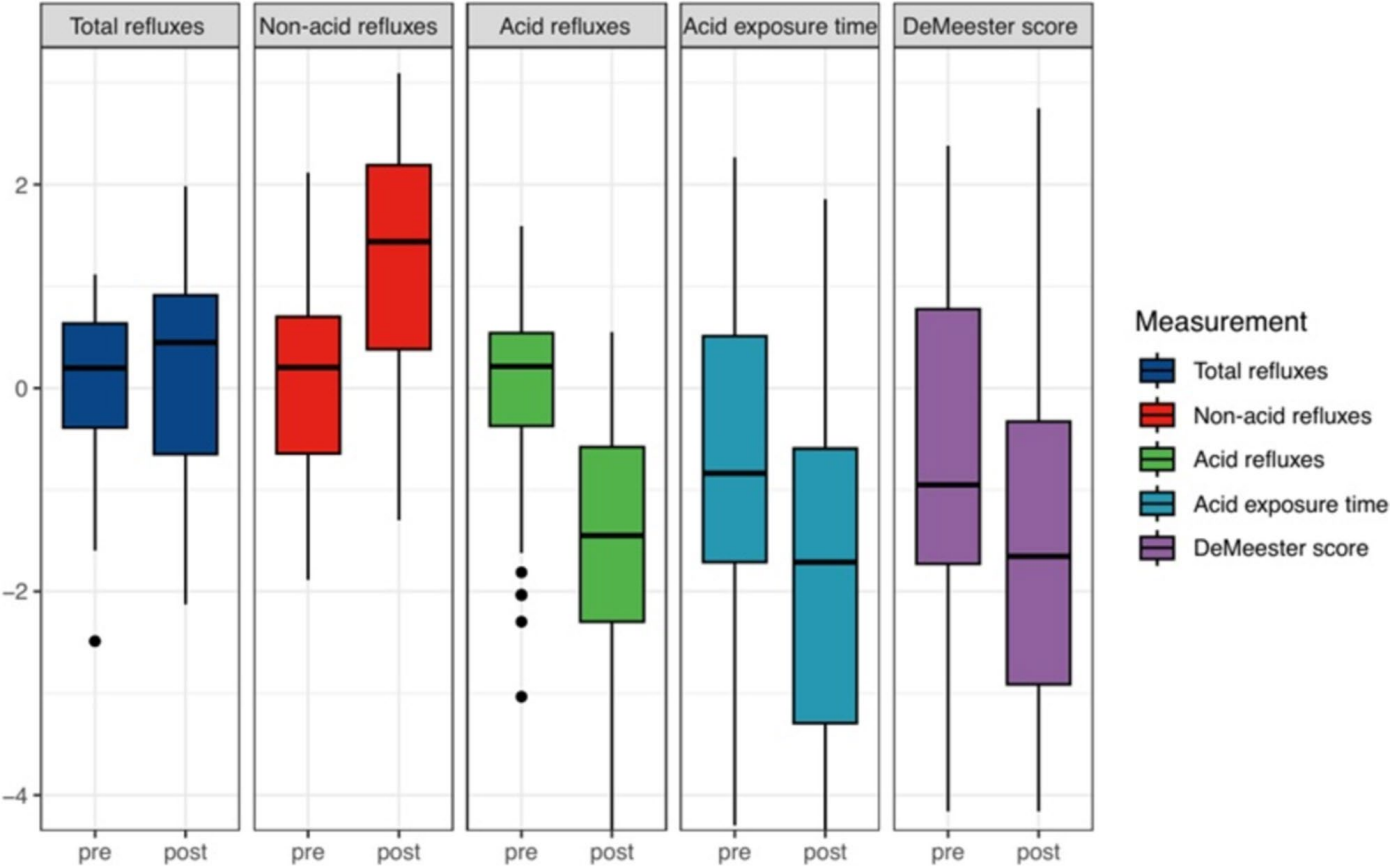
Impedance-24-h pH-metry before OAGB and at follow-up

The descriptive data in this study were presented as percentage or mean with standard deviation. Comparisons of functional testing before and after surgery were performed using Student’s *t* test with a *p* value of < 0.05 considered as significant. Figure 1 with the impedance-24-h pH-metry was created using the Wilcoxon test for paired samples. Further, SPSS® v28 for Windows® (IBM Corporation, Armonk, New York, USA) was used for statistical calculations.

## Results

This study includes all patients with gastroscopy, HRM, and impedance-24-h pH-metry before and after primary OAGB at the Medical University of Vienna. A total of 50 patients were willing to take part in these examinations at both time points. None of these patients had a previous operation before the OAGB. Five patients (10.0%) in this study received concomitant hiatal hernia repair at the time of the OAGB due to preoperative detection and intraoperative verification of the hiatal hernia. Anti-reflux sutures were performed in all patients. In this series, the majority of patients received a BPL of 150 cm and only a few patients a longer one of up to 250 cm. However, in this small series, no differences in the outcome with regard to the different BPL lengths were observed. Further baseline characteristics are presented in Table 1.

**Table 1:** Baseline characteristics and history of weight

	All patients
	(*n* = 50)
Sex (female) (*n* = 36)	72.0%
Age at OAGB (years)	43.9 ± 9.0
Previous bariatric procedures	0%
OAGB	
Length biliopancreatic limb (cm)	150–250
Hiatoplasty (*n* = 5)	10.0%
Anti-reflux sutures (*n* = 50)	100%
Weight at OAGB	
Weight (kg)	125.5 ± 21.0
BMI (kg/m^2^)	44.6 ± 5.4
Nadir weight	
Weight (kg)	74.2 ± 9.4
BMI (kg/m^2^)	26.9 ± 2.6
Change BMI	17.7 ± 3.1
TWL (%)	40.8 ± 8.2
EWL (%)	92.4 ± 15.2
Follow-up	
Minimal follow-up (years)	1.0
Mean follow-up (years)	4.1 ± 2.9
Median follow-up (years)	4.2 (R: 1.1–6.9)
Weight at follow-up	
Weight (kg)	79.0 ± 13.1
BMI (kg/m^2^)	28.7 ± 5.7
Change BMI	15.9 ± 4.6
TWL (%)	37.1 ± 8.1
EWL (%)	83.7 ± 17.1

*OAGB* one-anastomosis gastric bypass; *BMI* body mass index; *R* range

### Weight loss and remission of obesity-related complications

Mean weight and BMI at the time of the OAGB were 125.5 ± 21.0 kg and 44.6 ± 5.4 kg/m^2^. The lowest postoperative (nadir) weight and BMI these patients able to achieve were 74.2 ± 9.4 kg and 26.9 ± 2.6 kg/m^2^, which is equivalent to a total weight loss (TWL) of 40.8 ± 8.2%. Mean weight and BMI after the end of the follow-up period of 4.1 ± 2.9 years were 79.0 ± 13.1 kg and 28.7 ± 5.7, with a TWL of 37.1 ± 8.1%. Further weight loss results can be found in Table 1.

Remission of ORC within the follow-up period was observed as follows: Remission of T2D was noted in seven out of nine patients (77.8%), and of arterial hypertension in 14 out of 25 patients (56.0%). Additionally, three out of four patients (75%) with OSA and eight out of nine patients (88.8%) with hyperlipidemia experienced remission of their ORC. None of the patients in this study were suffering from GERD at the time of the OAGB. However, at the end of the follow-up period, six out of 50 (12.0%) were suffering from GERD and are currently successfully treated with proton pump inhibitors (PPI’s) for symptom control (Table 2).

**Table 2:** Obesity-related complications and GERD before OAGB and at follow-up

	All patients
Basis (OAGB)	(*n* = 50)
T2D	9 (18.0%)
Arterial hypertension	25 (50.0%)
OSA	4 (8.0%)
Hyperlipidemia	9 (18.0%)
GERD	0 (0%)

Follow-up	(*n* = 50)
T2D	2 (4.0%)
Arterial hypertension	11 (22.0%)
OSA	1 (2%)
Hyperlipidemia	1 (2%)
GERD	6 (12.0%)

*OAGB* one-anastomosis gastric bypass; *T2D* diabetes mellitus type 2; *OSA* obstructive sleep apnea; *GERD* gastro-esophageal reflux disease

### Gastroscopy

All patients in this study had gastroscopy before the OAGB and at the time of the follow-up of this study. In the preoperative gastroscopy, ten (20.0%) patients had antrum gastritis and seven (14.0%) patients mild esophagitis. In 14 patients (28.0%), a small hiatal hernia with a mean size of 1.62 cm (Range: 1–3 cm) was detected; however, after intraoperative visualization of both crura, only five of them were verified intraoperatively and repaired with hiatoplasty. *Helicobacter pylori* were eradicated (plus success verified) in six patients before the OAGB operation.

In the postoperative gastroscopy for this study, at the end of the follow-up period, pouchitis was detected in twelve patients (24.0%) and bile in the pouch in 24 patients (48.0%). However, none of these 24 patients had bile in the distal esophagus. The anastomositis rate was 28.0% (14 patients) macroscopically and 38.0% (19 patients) microscopically in the taken biopsies; eight of these patients also had asymptomatic anastomotic ulcers. The majority of them only had mild anastomosis. The esophagitis rate was 12.0% (six patients) macroscopic and 34.0% (17 patients) microscopic (biopsies). Interestingly, Barrett’s esophagus was detected in three patients (6.0%) who did not have it preoperatively. Further macroscopic and microscopic results of the preoperative and postoperative gastroscopies are presented in Table 3.

**Table 3:** Gastroscopy before OAGB and at follow-up

	All patients
Basis (OAGB)	(*n* = 50)
**Macroscopic**	
Gastritis	10 (20.0%)
Esophagitis	7 (14.0%)
Hiatal hernia	14 (28.0%)*
Mean hiatal hernia size	1.62 cm (R 1–3)
**Microscopic**	
*Helicobacter pylori*	6 (12.0%)**
Gastritis	12 (24.0%)
Esophagitis	6 (12.0%)
Barrett’s esophagus	0 (0%)

Follow-up	(*n* = 50)
**Macroscopic**	
Pouchitis	12 (24.0%)
Anastomositis	14 (28.0%)
Mild anastomositis	10 (20.0%)
Severe anastomositis	4 (8.0%)
Anastomotic ulcer	8 (16.0%)
Esophagitis	6 (12.0%)
Hiatal hernia	0 (0.0%)***
Bile in the pouch	24 (48.0%)
**Microscopic**	
*Helicobacter pylori*	1 (2.0%)
Pouchitis	9 (18%)
Anastomositis	19 (38.0%)
Mild anastomositis	14 (28.0)
Severe anastomositis	5 (10.0)
Esophagitis	17 (34.0%)
Barrett’s esophagus	3 (6.0%)

*OAGB* one-anastomosis gastric bypass; *R* range

*All patients with hiatal hernia in the preoperative gastroscopy were explored intraoperatively and a hiatoplasty was performed if indicated

**Helicobacter pylori infections were eradicated preoperatively and verified by stool test

***Hiatal hernias are hard to detect in patients with a proper pouch where inversion with the gastroscope is not possible

### High-resolution manometry and impedance-24-h pH-metry

Comparing data gained from HRM before and 4.1 ± 2.9 years after primary OAGB, one can see that LESP did not change significantly: 27.7 ± 12.3 vs. 29.6 ± 15.1 mmHg (*p* = 0.439). The time of a swallowed liquid bolus transferred through the esophagus to the stomach/pouch decreased significantly from 6.2 ± 1.9 to 4.6 ± 2.1 s (*p* = 0.001), both values are within the normal range.

Comparing the results of the impedance-24-h pH-metry before and 5.1 years after primary OAGB, acid exposure time of the esophagus decreases significantly from 4.4 ± 4.2 to only 1.6 ± 1.4%; *p* = 0.001. Further, the total number of refluxes in 24 h into the esophagus increased only slightly (not statistically significant) from 46.6 ± 19.5 (preoperatively) to 56.6 ± 34.8 (at follow-up), but the composition has changed. While the number of non-acid refluxes nearly tripled (18.7 ± 12.6 to 46.3 ± 31.0; *p* = 0.001), the number of acid refluxes more than halved (27.9 ± 17.7 to only 10.3 ± 9.5; *p* = 0.001). The DeMeester score, which mainly reflects acid reflux, also decreased significantly from 17.8 ± 16.7 into the normal range of 10.3 ± 9.6; *p* = 0.046. Further results of functional esophageal testing can be seen in Table 4 and Fig. 1.

**Table 4:** Functional testing (HRM and impedance-24-h pH-metry) before OAGB and at follow-up

	All patients		
	Basis OAGB	Follow-up	*p* value
	(*n* = 50)	(*n* = 50)	
Manometry			
LESP (mmHg) (10–35 mmHg)	27.7 ± 12.3	29.6 ± 15.1	0.439
Time liquid bolus (s) (< 12 s)	6.2 ± 1.9	4.6 ± 2.1	**0.001**
IRP (mmHg) (< 15 mmHg)	12.7 ± 6.2	12.0 ± 6.7	0.501
DCI (mmHg-cm-s) (450–8000 mmHg-cm-s)	2270.0 ± 1539.8	1612.7 ± 1067.2	**0.017**
Impedance 24 h-pH-metry			
Acid exposure time (% of 24 h) (normal < 4.2%)	4.4 ± 4.2	1.6 ± 1.4	**0.001**
Total number of refluxes (normal < 40)	46.6 ± 19.5	56.6 ± 34.8	0.060
Number non-acid refluxes	18.7 ± 12.6	46.3 ± 31.0	**0.001**
Number acid refluxes	27.9 ± 17.7	10.3 ± 9.5	**0.001**
DeMeester score (normal 14.72)	17.8 ± 16.7	10.3 ± 9.6	**0.046**

Bold values are statistically signifcant

*OAGB* one-anastomosis gastric bypass; *HRM* high-resolution manometry; *LESP* lower esophageal sphincter pressure; *IRP* integrated relaxation pressure; *DCI* distal contractile integral; *s* seconds

### Quality of life

The results of the SF-36 questionnaire in the eight categories are displayed in Table 5. In BQL and GIQLI, patients were able to reach 53.9 ± 9.6 and 111.2 ± 20.7 points, respectively. The score of BAROS was 5.2 ± 1.8, which equals a “very good” outcome after MBS (the second best rating) in the corresponding key.

**Table 5:** Quality of life and bariatric surgery outcome before OAGB and at follow-up

Follow-up	All patients
	(*n* = 34)
SF-36	
Physical functioning (PF)	87.6 ± 15.7
Role physical (RP)	87.1 ± 25.7
Bodily pain (BP)	75.3 ± 25.1
General health (GH)	75.8 ± 19.8
Vitality (VT)	65.5 ± 23.5
Social functioning (SF)	86.4 ± 20.1
Role emotional (RE)	90.9 ± 24.0
Mental health (MH)	79.6 ± 17.2
BQL	53.9 ± 9.6
GIQLI	111.2 ± 20.7
BAROS (with comorbidities)	5.2 ± 1.8

*SF-36* Short Form-36; *BQL* Bariatric Quality of Life index; *GIQLI* Gastrointestinal Quality of Life Index; *BAROS* Bariatric Analysis and Reporting Outcome System

## Discussion

This study reflects a mid-term outcome after OAGB, based on different objective examinations. The advantage of this study over other studies available at this time is that results of gastroscopy, HRM, and impedance-24-h pH-metry were not only available after but also before the primary OAGB. Comparing pre-and postoperative results, increasing acid and decreasing non-acid (biliary) refluxes were found. Further, several interesting findings in the postoperative endoscopy were examined. In terms of weight loss, remission of ORC, and QOL, excellent mid-term results were achieved.

This study is an update to the previously published study with 21 patients, now including a total of 50 patients [17]. The 21 original patients are included in the present study and were not re-examined postoperative for this update. Due to the fact that the number of included patients were more than doubled in the follow-up study, the validity of the presented data has increased significantly. Motivating patients to participate in these relatively uncomfortable follow-up examinations was possible with a financial incentive to them, using the EAES research grant.

### Weight loss and remission of obesity-related complications

Regarding weight loss, the outcome in this study can be seen as very successful. After a mean mid-term follow-up of 4.1 years, TWL was still more than 35% and EWL over 80%. Thus, at the end of follow-up, the majority of patients is now “overweight” (BMI between 25 and 30 kg/m^2^) instead of “severely obese” as before, which leads to more comfortability without a huge risk of malnutrition. Also, comparing pre- and postoperative ORC’s, the procedure may be termed a success (with over 75% remission rates for T2D, OSA, and hyperlipidemia). Even the remission rate of arterial hypertension at more than 50% may be seen as successful.

In this study, a few patients with a BPL of 200–250 cm were included. However, we have changed the technique since then to doing 150-cm BPL due to a higher risk of malnutrition without a significant additional effect on weight loss and remission of ORC in the literature [26] and in our own experience [27]. Now, our standard BPL length is 150 cm and will not be increased to more than 175 cm, which is in line with the safety considerations of IFSO [26].

Recently published 5-year results of the YOMEGA study (a randomized controlled trial (RCT) comparing OAGB to RYGB) by Robert et al. found an EWL of 75.6% after 5 years and T2D remission rate of 58.0% after OAGB [28]. Another study by Van der Laan et al. with 860 OAGB patients also showed a TWL of 30.0%, EWL of 74.0%, and remission rates for T2D and arterial hypertension of more than 60% 5 years after OAGB [29]. In summary, weight loss and remission of ORC results in this study are in line with the current literature.

### Acid/biliary reflux

Biliary reflux is one of the “hot topics” when discussing the advantages and disadvantages of OAGB. Nevertheless, most studies do not distinguish between acid and non-acid reflux. Furthermore, the majority of studies dealing with this topic only describe the need of a conversion to RYGB without any prior objective measurement. Therefore, no final conclusion can be drawn if those patients did in fact suffer from acid or non-acid (biliary) reflux. The conversion rate in these studies varies from 0.4 to 7.6% [12, 30]. The conversion rate from OAGB to RYGB due to symptomatic reflux in our bariatric center was 4.2% [27]. Interestingly, a blockage of drainage in the common limb was observed intraoperatively in a large number of patients caused, e.g., by kinking of the small bowel, an adhesion, or a Petersen’s space hernia [27]. Therefore, the authors suggest checking the entire small bowel when doing conversions from OAGB to RYGB due to reflux.

Gastroscopy, used in this study as an additional examination, has its advantages and disadvantages. It is almost immediately available in most bariatric centers and easy to perform. On the other hand, only the effects of reflux, such as esophagitis, pouchitis, anastomositis, Barrett’s esophagus, and ulcers, can be detected but it is difficult to distinguish between acid, non-acid (biliary), and mixed reflux. Furthermore, gastroscopy is basically a snapshot examination with the patient in a lateral position. Bile in the pouch/esophagus may thus be overestimated and gastroscopy does not indicate under which circumstances the reflux occurs (at all times, during the night time when sleeping, after food intake, etc.).

In a study by Pizza et al., 241 patients were examined 12 months after OAGB with endoscopy and GERD health-related QOL. The results showed an increased incidence of esophagitis but lower than in the current study. This could be due to the shorter follow-up as the irritation of the tissue/lesions will probably increase over time [31]. Saarinen et al. studied the outcome 6 months after OAGB by performing gastroscopies and scintigraphy. In 39.5% of their patients, suspicious findings such as esophagitis, ulcers, and inflammation of the pouch were found. The results of the scintigraphy were biliary reflux up to the pouch in 31.6% of their patients [32]. These endoscopic findings are similar to those of the presented study. Similarly, scintigraphy results in terms of bile in the pouch are comparable to endoscopic results of the present study as well. It is important to mention here that the detection of biliary reflux by scintigraphy is superior to endoscopy as the tracer is specifically linked to bile and the examination period is much longer at 60–120 min [32]. Another study by Arnon-Sheleg et al. found biliary reflux to the pouch in 53.5% and to the esophagus in 21.0% of their patients at 22 months after OAGB [33]. However, it should be noted that the authors did not use impedance 24 h-pH-metry, which is the only way to gather information about reflux episodes during an entire day (24 h).

The rate of ulcers found in the gastroscopy was rather high in this study, nevertheless, the majority were asymptomatic and clinically not relevant. To further explore this topic, more studies are needed that systematically do endoscopy on OAGB patients in the follow-up, regardless of symptoms.

Impedance 24 h-pH-metry might be the tool with the highest potential for the detection of reflux and the differentiation between acid and non-acid fluid. It makes sense to combine this examination with HRM for adding information about the swallowing act, esophagus motility, and the LES. Impedance 24 h-pH-metry cannot only count the number of refluxes and measure their pH value but also records the length of the episodes. Plus, the circumstances (eating, sleeping, sport, etc.) of each reflux episode can be evaluated if an additional diary is filled in by the patient.

The current study showed that acid reflux decreases after OAGB, which seems logical as the pylorus is not in the food stream anymore and acid can easily drain into the common limb (low pressure system). On the other hand, the physiological anatomy of the pylorus also has the purpose to prevent bile from flowing up to the pouch/esophagus. Therefore, increasing non-acid/biliary reflux episodes were detected, especially in supine position. It must be mentioned here that both examinations take place in the esophagus and not in the pouch, which means that bile can also pass the LES upward, even though the LESP was normal in almost all patients in this study. Comparing LESP pre- and postoperatively and finding it unchanged (normal), is also a good indicator that the LES was not harmed during the bypass procedure.

In this study, the DCI decreased significantly when comparing pre- to postoperative results, but remained within the normal range (in all patients) with a great safety distance to the normal lower level of 450 mmHg-cm-s. Nevertheless, as no additional postoperative follow-up examinations are available, this study cannot provide information on whether the decrease of DCI would continue over time and if acid or non-acid reflux episodes have any influence on that. In a study by Matar et al. where postoperative Sleeve Gastrectomy patients were measured with HRM and compared to a control group, a connection between lower DCI levels and GERD symptoms were found [34]. Further studies on this topic are rare, which makes it an important topic for future research.

Comparing our results to the literature, Nemeth et al. also performed 24-h pH-metry 64 months after OAGB in 43 patients (no preoperative data) and found 30.2% of their patients had acid reflux and 27.9% had biliary reflux. While the rate of biliary reflux is equal to the present study’s results, acid reflux was much higher, which may be due to by the fact that Nemeth et al. included revisional patients (gastric banding) and patients with preoperative reflux in their study [15]. Such patients would currently receive a RYGB in our metabolic/bariatric center. A smaller case series by Tolone et al. with a shorter follow-up of 1 year also found decreasing acid and decreasing non-acid reflux when comparing pre- and postoperative results. This difference can probably be explained by the shorter follow-up, evaluated at a time when the intraabdominal pressure is on its lowest point [16]. Another small study with eleven patients by Doulami et al. and a similar design found a slight increase in non-acid reflux and a decrease in acid reflux, in line with the current study [35].

All these facts lead to the question of whether biliary reflux is merely an uncomfortable side effect or if it has serious consequences. Li et al. studied the connection of biliary reflux and gastric cancer in over 30.000 patients that underwent gastroscopy and found that biliary reflux is associated with precancerous lesions [36]. Further, Hong et al. studied Barrett cells and found that bile acid can induce DNA damage and lead to esophageal adenocarcinoma [37].

The described anti-reflux sutures that were performed in all patients in the present study were first described by Carbajo [38] in 2004. A retrospective study by Slagter et al. compared 414 patients with and 289 patients without anti-reflux sutures. While no differences in clinical symptoms were observed, the conversion rate to RYGB was significantly lower in the group with anti-reflux sutures [39]. A current RCT by Kermansaravri et al. comparing 50 patients with anti-reflux sutures to 50 patients without found lower de novo GERD and higher GERD remission rates in the group with anti-reflux sutures 1 year after the OAGB [40].

To sum up, data on objective functional esophageal testing is currently only available based on a few patients after OAGB. In any case, it seems to be important to differentiate between revisional and non-revisional patients, short-, mid-, and long-term follow-up, as well as between procedures with and without anti-reflux sutures when comparing data. Further, it would be very interesting to see if the results of Impedance-24-h pH-metry and HRM change over time postoperatively.

### Outcome and quality of life

The different QOL and outcome scores used in this study reflect different categories of patient comfort. Unfortunately, these test results were not available in this cohort from before the OAGB operation, therefore the scores can only be compared to the literature.

Comparing the present QOL scores to a currently published series of QOL after Sleeve Gastrectomy from the authors of the same bariatric center, OAGB was superior in all categories of SF-36, BQL, and GIQLI. Further, also the objective BAROS indicates a much better outcome after OAGB than after Sleeve Gastrectomy [41–43]. In another comparative study by Madani et al. with 299 patients, OAGB also scored significantly higher than Sleeve Gastrectomy or RYGB after 5 years in the BAROS [44].

Another study comparing QOL in OAGB to RYGB before and 2 years after the operations with different tools (SF-34 and BAROS) has also shown a significant improvement from pre- to postoperative scores. However, no difference was seen between the OAGB and RYGB procedures [45]. It has to be considered here that the follow-up was much shorter (2 years) than in the present study as well as the other studies discussed above. Further, Singla et al. reported the gastrointestinal QOL with a mini GIQLI after 3 and 5 years and found high QOL scores comparable to the ones in the current study [46]. In conclusion, QOL in a mid-term follow-up after OAGB seems to be relatively high (also compared to other bariatric procedures).

### Limitations of this study

A limitation of this study is the small number of patients, even though we were able to more than double the number of patients in this follow-up study compared to the study previously published. Unfortunately, esophageal functional testing involves uncomfortable examinations that not all patients are willing to take part in. This, of course, may mean a possible risk of selection bias as patients with GERD symptoms may be more willing to do esophageal functional testing another time over. Further, 50 out of 969 primary OAGB patients may not be representative of the entire patients collective. Another point is that patients had the examinations for this study at different individual postoperative timepoints, which may affect the results. Additionally, patients that had hiatoplasty at the time of the OAGB could have influenced the outcome of this study. On the other hand, this reflects a normal collective of patients with severe obesity.

Another limitation of this study is that the QOL questionnaires were not performed preoperatively, before the OAGB, so a comparison of pre- to the postoperative scores was not possible. Regardless, this QOL data could be compared to the literature.

## Conclusion

This study has shown increased rates of non-acid reflux and decreased rates of acid -reflux after a mid-term follow-up in primary OAGB patients. Additionally, in gastroscopy, signs of chronic irritation of the gastro-jejunostomy, the distal esophagus, the pouch, and the (mild) anastomosis and (asymptomatic) ulcers were found, even in a majority of asymptomatic patients. Thus, general follow-up gastroscopies in OAGB patients after 5 years should be considered. Regarding weight loss, remission of ORC, and QOL, the results of this study were quite good.

## References

[1] Gensthaler L, Felsenreich DM, Jedamzik J, Eichelter J, Nixdorf L, Bichler C, Krebs M, Itariu B, Langer FB, Prager G (2022) Trends of overweight and obesity in male adolescents: prevalence, socioeconomic status, and impact on cardiovascular risk in a Central European Country. Obes Surg. 10.1007/s11695-021-05867-z 35041124 10.1007/s11695-021-05867-z PMC8933384

[2] Liu N, Birstler J, Venkatesh M, Hanrahan L, Chen G, Funk L (2021) Obesity and BMI cut points for associated comorbidities: electronic health record study. J Med Internet Res 23:e24017 34383661 10.2196/24017 PMC8386370

[3] Carlsson LMS, Sjoholm K, Jacobson P, Andersson-Assarsson JC, Svensson PA, Taube M, Carlsson B, Peltonen M (2020) Life expectancy after bariatric surgery in the Swedish Obese Subjects Study. N Engl J Med 383:1535–1543 33053284 10.1056/NEJMoa2002449 PMC7580786

[4] Abiri B, Hosseinpanah F, Banihashem S, Madinehzad SA, Valizadeh M (2022) Mental health and quality of life in different obesity phenotypes: a systematic review. Health Qual Life Outcomes 20:63 35439997 10.1186/s12955-022-01974-2 PMC9019986

[5] Jarvholm K, Janson A, Peltonen M, Neovius M, Gronowitz E, Engstrom M, Laurenius A, Beamish AJ, Dahlgren J, Sjogren L, Olbers T (2023) Metabolic and bariatric surgery versus intensive non-surgical treatment for adolescents with severe obesity (AMOS2): a multicentre, randomised, controlled trial in Sweden. Lancet Child Adolesc Health 7:249–260 36848922 10.1016/S2352-4642(22)00373-X

[6] Angrisani L, Santonicola A, Iovino P, Ramos A, Shikora S, Kow L (2021) Bariatric surgery survey 2018: similarities and disparities among the 5 IFSO chapters. Obes Surg 31:1937–1948 33432483 10.1007/s11695-020-05207-7 PMC7800839

[7] Ramos AC, Chevallier JM, Mahawar K, Brown W, Kow L, White KP, Shikora S, Contributors ICC (2020) IFSO (International Federation for Surgery of Obesity and Metabolic Disorders) consensus conference statement on one-anastomosis gastric bypass (OAGB-MGB): results of a modified Delphi Study. Obes Surg 30:1625–1634 32152841 10.1007/s11695-020-04519-y

[8] Felsenreich DM, Bichler C, Langer FB, Gachabayov M, Eichelter J, Gensthaler L, Vock N, Artemiou E, Prager G (2020) Surgical technique for one-anastomosis gastric bypass. Surg Technol Int 37:57–61 33180956

[9] Felsenreich DM, Langer FB, Bichler C, Eichelter J, Jedamzik J, Gachabayov M, Mairinger M, Richwien P, Prager G (2021) Surgical technique of diverted one-anastomosis gastric bypass. Surg Technol Int 39:107–112 34699605

[10] Ahuja A, Tantia O, Goyal G, Chaudhuri T, Khanna S, Poddar A, Gupta S, Majumdar K (2018) MGB-OAGB: effect of biliopancreatic limb length on nutritional deficiency, weight loss, and comorbidity resolution. Obes Surg 28:3439–3445 30032419 10.1007/s11695-018-3405-7

[11] Chevallier JM, Arman GA, Guenzi M, Rau C, Bruzzi M, Beaupel N, Zinzindohoue F, Berger A (2015) One thousand single anastomosis (omega loop) gastric bypasses to treat morbid obesity in a 7-year period: outcomes show few complications and good efficacy. Obes Surg 25:951–958 25585612 10.1007/s11695-014-1552-z

[12] Liagre A, Debs T, Kassir R, Ledit A, Juglard G, Chalret du Rieu M, Lazzati A, Martini F, Petrucciani N (2020) One anastomosis gastric bypass with a biliopancreatic limb of 150 cm: weight loss, nutritional outcomes, endoscopic results, and quality of life at 8-year follow-up. Obes Surg 30:4206–4217 32562132 10.1007/s11695-020-04775-y

[13] Musella M, Susa A, Greco F, De Luca M, Manno E, Di Stefano C, Milone M, Bonfanti R, Segato G, Antonino A, Piazza L (2014) The laparoscopic mini-gastric bypass: the Italian experience: outcomes from 974 consecutive cases in a multicenter review. Surg Endosc 28:156–163 23982648 10.1007/s00464-013-3141-y

[14] Kular KS, Manchanda N, Rutledge R (2014) A 6-year experience with 1,054 mini-gastric bypasses-first study from Indian subcontinent. Obes Surg 24:1430–1435 24682767 10.1007/s11695-014-1220-3

[15] Nehmeh WA, Baratte C, Rives-Lange C, Martineau C, Boullenois H, Krivan S, Guillet V, Le Gall M, Cellier C, Carette C, Czernichow S, Chevallier JM, Poghosyan T (2021) Acid reflux is common in patients with gastroesophageal reflux disease after one-anastomosis gastric bypass. Obes Surg 31:4717–4723 34232446 10.1007/s11695-021-05542-3

[16] Tolone S, Cristiano S, Savarino E, Lucido FS, Fico DI, Docimo L (2016) Effects of omega-loop bypass on esophagogastric junction function. Surg Obes Relat Dis 12:62–69 25979206 10.1016/j.soard.2015.03.011

[17] Felsenreich DM, Zach ML, Vock N, Jedamzik J, Eichelter J, Mairinger M, Gensthaler L, Nixdorf L, Richwien P, Bichler C, Kristo I, Langer FB, Prager G (2023) Esophageal function and non-acid reflux evaluated by impedance-24 h-pH-metry, high-resolution manometry, and gastroscopy after one-anastomosis gastric bypass-outcomes of a prospective mid-term study. Surg Endosc 37:3832–3841 36693919 10.1007/s00464-022-09857-9 PMC10156623

[18] Levine DS, Haggitt RC, Blount PL, Rabinovitch PS, Rusch VW, Reid BJ (1993) An endoscopic biopsy protocol can differentiate high-grade dysplasia from early adenocarcinoma in Barrett’s esophagus. Gastroenterology 105:40–50 8514061 10.1016/0016-5085(93)90008-z

[19] American Gastroenterological A, Spechler SJ, Sharma P, Souza RF, Inadomi JM, Shaheen NJ (2011) American Gastroenterological Association medical position statement on the management of Barrett’s esophagus. Gastroenterology 140:1084–1091 21376940 10.1053/j.gastro.2011.01.030

[20] Yadlapati R (2017) High-resolution esophageal manometry: interpretation in clinical practice. Curr Opin Gastroenterol 33:301–309 28426462 10.1097/MOG.0000000000000369 PMC5568812

[21] Kristo I, Paireder M, Jomrich G, Felsenreich DM, Nikolic M, Langer FB, Prager G, Schoppmann SF (2019) Modern esophageal function testing and gastroesophageal reflux disease in morbidly obese patients. Obes Surg 29:3536–3541 31201693 10.1007/s11695-019-04020-1

[22] Ware JE Jr, Sherbourne CD (1992) The MOS 36-item short-form health survey (SF-36). I. Conceptual framework and item selection. Med Care 30:473–483 1593914

[23] Eypasch E, Wood-Dauphinee S, Williams JI, Ure B, Neugebauer E, Troidl H (1993) The Gastrointestinal Quality of Life Index. A clinical index for measuring patient status in gastroenterologic surgery. Chirurg 64:264–274 8482141

[24] Weiner S, Sauerland S, Weiner R, Cyzewski M, Brandt J, Neugebauer E (2009) Validation of the adapted Bariatric Quality of Life Index (BQL) in a prospective study in 446 bariatric patients as one-factor model. Obes Facts 2(Suppl 1):63–66 20124782 10.1159/000198263 PMC6444798

[25] Oria HE, Moorehead MK (1998) Bariatric analysis and reporting outcome system (BAROS). Obes Surg 8:487–499 9819079 10.1381/096089298765554043

[26] De Luca M, Piatto G, Merola G, Himpens J, Chevallier JM, Carbajo MA, Mahawar K, Sartori A, Clemente N, Herrera M, Higa K, Brown WA, Shikora S (2021) IFSO update position statement on one anastomosis gastric bypass (OAGB). Obes Surg 31:3251–3278 33939059 10.1007/s11695-021-05413-x

[27] Jedamzik J, Bichler C, Felsenreich DM, Gensthaler L, Eichelter J, Nixdorf L, Krebs M, Langer FB, Prager G (2022) Conversion from one-anastomosis gastric bypass to Roux-en-Y gastric bypass: when and why-a single-center experience of all consecutive OAGB procedures. Surg Obes Relat Dis 18:225–232 34794865 10.1016/j.soard.2021.10.019

[28] Robert M, Poghosyan T, Maucort-Boulch D, Filippello A, Caiazzo R, Sterkers A, Khamphommala L, Reche F, Malherbe V, Torcivia A, Saber T, Delaunay D, Langlois-Jacques C, Suffisseau A, Bin S, Disse E, Pattou F (2024) Efficacy and safety of one anastomosis gastric bypass versus Roux-en-Y gastric bypass at 5 years (YOMEGA): a prospective, open-label, non-inferiority, randomised extension study. Lancet Diabetes Endocrinol 12:267–276 38452784 10.1016/S2213-8587(24)00035-4

[29] van der Laan L, Sizoo D, van Beek AP, Emous M, Dutch Audit for Treament of Obesity Research G (2024) Comparable results 5 years after one anastomosis gastric bypass compared to Roux-en-Y gastric bypass: a propensity-score matched analysis. Surg Obes Relat Dis. 10.1016/j.soard.2024.09.009 39472258 10.1016/j.soard.2024.09.009

[30] Noun R, Skaff J, Riachi E, Daher R, Antoun NA, Nasr M (2012) One thousand consecutive mini-gastric bypass: short- and long-term outcome. Obes Surg 22:697–703 22411569 10.1007/s11695-012-0618-z

[31] Pizza F, D’Antonio D, Lucido FS, Tolone S, Dell’Isola C, Gambardella C (2020) Postoperative clinical-endoscopic follow-up for GERD and gastritis after one anastomosis gastric bypass for morbid obesity: how, when, and why. Obes Surg 30:4391–4400 32621053 10.1007/s11695-020-04805-9

[32] Saarinen T, Pietilainen KH, Loimaala A, Ihalainen T, Sammalkorpi H, Penttila A, Juuti A (2020) Bile reflux is a common finding in the gastric pouch after one anastomosis gastric bypass. Obes Surg 30:875–881 31853864 10.1007/s11695-019-04353-x PMC7347680

[33] Arnon-Sheleg E, Farraj M, Michael S, Mari A, Khoury T, Sbeit W (2023) Modified hepatobiliary scintigraphy for the diagnosis of bile reflux in one-anastomosis gastric bypass surgery: a prospective multicenter study. Obes Surg 33:1997–2004 37184825 10.1007/s11695-023-06632-0

[34] Matar R, Maselli D, Vargas E, Veeravich J, Bazerbachi F, Beran A, Storm AC, Kellogg T, Abu Dayyeh BK (2020) Esophagitis after bariatric surgery: large cross-sectional assessment of an endoscopic database. Obes Surg 30:161–168 31584148 10.1007/s11695-019-04164-0

[35] Doulami G, Triantafyllou S, Albanopoulos K, Natoudi M, Zografos G, Theodorou D (2018) Acid and nonacid gastroesophageal reflux after single anastomosis gastric bypass. An objective assessment using 24-hour multichannel intraluminal impedance-pH metry. Surg Obes Relat Dis 14:484–488 29203406 10.1016/j.soard.2017.10.012

[36] Li D, Zhang J, Yao WZ, Zhang DL, Feng CC, He Q, Lv HH, Cao YP, Wang J, Qi Y, Wu SR, Wang N, Zhao J, Shi YQ (2020) The relationship between gastric cancer, its precancerous lesions and bile reflux: a retrospective study. J Dig Dis 21:222–229 32187838 10.1111/1751-2980.12858 PMC7317534

[37] Hong J, Behar J, Wands J, Resnick M, Wang LJ, DeLellis RA, Lambeth D, Souza RF, Spechler SJ, Cao W (2010) Role of a novel bile acid receptor TGR5 in the development of oesophageal adenocarcinoma. Gut 59:170–180 19926617 10.1136/gut.2009.188375 PMC3049934

[38] Garcia-Caballero M, Carbajo M (2004) One anastomosis gastric bypass: a simple, safe and efficient surgical procedure for treating morbid obesity. Nutr Hosp 19:372–375 15672654

[39] Slagter N, Hopman J, Altenburg AG, de Heide LJM, Jutte EH, Kaijser MA, Damen SL, van Beek AP, Emous M (2021) Applying an anti-reflux suture in the one anastomosis gastric bypass to prevent biliary reflux: a long-term observational study. Obes Surg 31:2144–2152 33496931 10.1007/s11695-021-05238-8

[40] Kermansaravi M, Shahsavan M, Ebrahimi R, Mousavimaleki A, Gholizadeh B, Valizadeh R, ShahabiShahmiri S, Carbajo MA (2024) Effect of anti-reflux suture on gastroesophageal reflux symptoms after one anastomosis gastric bypass: a randomized controlled trial. Surg Endosc 38:2562–2570 38499781 10.1007/s00464-024-10792-0

[41] Felsenreich DM, Artemiou E, Steinlechner K, Vock N, Jedamzik J, Eichelter J, Gensthaler L, Bichler C, Sperker C, Beckerhinn P, Kristo I, Langer FB, Prager G (2021) Fifteen years after sleeve gastrectomy: weight loss, remission of associated medical problems, quality of life, and conversions to Roux-en-Y gastric bypass-long-term follow-up in a multicenter study. Obes Surg 31:3453–3461 34021882 10.1007/s11695-021-05475-x PMC8270807

[42] Felsenreich DM, Artemiou E, Wintersteller L, Jedamzik J, Eichelter J, Gensthaler L, Bichler C, Sperker C, Beckerhinn P, Kristo I, Langer FB, Prager G (2022) Fifteen years after sleeve gastrectomy: gastroscopies, manometries, and 24-h pH-metries in a long-term follow-up: a multicenter study. Obes Facts 15:666–673 35882187 10.1159/000526170 PMC9669976

[43] Felsenreich DM, Prager G, Kefurt R, Eilenberg M, Jedamzik J, Beckerhinn P, Bichler C, Sperker C, Krebs M, Langer FB (2019) Quality of life 10 years after sleeve gastrectomy: a multicenter study. Obes Facts 12:157–166 30879011 10.1159/000496296 PMC6547272

[44] Madani S, Shahsavan M, Pazouki A, Setarehdan SA, Yarigholi F, Eghbali F, Shahmiri SS, Kermansaravi M (2024) Five-year BAROS score outcomes for Roux-en-Y gastric bypass, one anastomosis gastric bypass, and sleeve gastrectomy: a comparative study. Obes Surg 34:487–493 38147191 10.1007/s11695-023-07015-1

[45] Rheinwalt KP, Fobbe A, Plamper A, Alizai PH, Schmitz SM, Brol MJ, Trebicka J, Neumann UP, Ulmer TF (2023) Health-related quality of life outcomes following Roux-en-Y gastric bypass versus one anastomosis gastric bypass. Langenbecks Arch Surg 408:74 36729181 10.1007/s00423-023-02792-w

[46] Singla V, Kumar A, Gupta M, Manohar M, Monga S, Agarwal S, Sharma AK, Aggarwal S (2022) Gastrointestinal quality of life in morbidly obese patients undergoing one anastomosis gastric bypass (OAGB): derivation of a “Mini GIQLI” score. Obes Surg 32:2332–2340 35488108 10.1007/s11695-022-06080-2

